# Supplementary material for: Investigation of the global translational response to oxidative stress in the model archaeon Haloferax volcanii reveals untranslated small RNAs with ribosome occupancy
Source: mSphere. 2025 Sep 8;10(9):e00343-25. doi: 10.1128/msphere.00343-25 (PMC12482152; doi:10.1128/msphere.00343-25)
Supplement: Supplemental Material — Supplemental tables and figures. [file msphere.00343-25-s0001.pdf]

SUPPLEMENTARY MATERIAL

Investigation of the global translational response to oxidative stress in the model archaeon *Haloferax volcanii* reveals untranslated small RNAs with ribosome occupancy

Emma Dallon, Haley Moran, Sadhana R. Chidambaran, Arman Kian, Betty Y.H. Huang, Stephen Fried, Jocelyne DiRuggiero*

TABLES S1 TO S23

Table S1. TPMs calculated from RNA-seq data. See xlsx file.

Table S2. TPMS calculated from Ribo-seq data. See xlsx file.

Table S3. Differential expression values for RNA-seq and Ribo-seq comparing stress and no stress conditions. See xlsx file.

Table S4. Differential expression values for RNA-seq and Ribo-seq for genes previously shown to be involved in oxidative stress response.

Gene name	Description	RNA-seq		Ribo-seq	
		log_2 fold change	p-value	log_2 fold change	p-value
**HVO_RS03960**	Superoxide dismutase	1.82	9.35E-11	2.35	3.88E-11
**HVO_RS13250**	Catalase	2.48	2.92E-27	1.49	1.34E-10
**HVO_RS06755**	Universal stress protein	1.37	1.89E-07	1.84	9.93E-12
**HVO_RS06110**	ssDNA binding replication protein	3.21	9.87E-36	3.09	2.71E-21
**HVO_RS07285**	stationary phase protection protein	2.79	3.95E-21	1.18	0.001
**HVO_RS00195**	IucA/IucC family siderophore biosynthesis protein	-3.59	6.54E-17	-3.54	0.002
**HVO_RS00200**	lysine N(6)-hydroxylase/L-ornithine N(5)-oxygenase family protein	-3.78	4.43E-23	-3.39	0.002
**HVO_RS00205**	GNAT family N-acetyltransferase	-3.19	4.06E-18	-3.20	0.003
**HVO_RS00210**	IucA/IucC family protein	-3.33	2.66E-15	-3.33	0.002
**HVO_RS07290**	metal-dependent transcriptional regulator	-1.99	6.04E-13	-1.32	0.007

Table S5. Characteristics for the whole genome, and for genes with differential translation (TE), ribosome occupancy (Ribo-seq), and expression (RNA-seq) levels.

	% leadered	% upregulated	% downregulated	Size range (nt)	Average size (nt)
**RNA-seq**	18.75	56.2	43.8	27-4536	776
**Ribo-seq**	22.84	43.1	56.9	27-4536	773
**TE**	30	29.9	70.1	42-2850	717
**Whole genome**	20	-	-	27-6717	823

Table S6. Functional categories assigned by PANTHER. See xlsx file.

Table S7. Sample sizes and percentage values for functional enrichment of genes with differential TE.

	full proteome		TE down			TE up		
protein class	count	percent	count	percent	enrichment	count	percent	enrichment
cell adhesion molecule	1	0.00	0	0.00	0.00	0	0.00	0.00
chaperone	29	0.01	6	0.06	4.31	0	0.00	0.00
chromatin	9	0.00	0	0.00	0.00	0	0.00	0.00
cytoskeletal protein	7	0.00	0	0.00	0.00	0	0.00	0.00
defense/immunity protein	3	0.00	0	0.00	0.00	0	0.00	0.00
DNA metabolism protein	104	0.05	2	0.02	0.40	0	0.00	0.00
gene-specific transcriptional regulator	189	0.09	9	0.09	0.99	4	0.10	1.04
membrane traffic protein	6	0.00	1	0.01	3.47	0	0.00	0.00
metabolite interconversion enzyme	971	0.47	46	0.46	0.99	15	0.36	0.76
protein modifying enzyme	81	0.04	1	0.01	0.26	5	0.12	3.03
protein-binding activity modulator	15	0.01	3	0.03	4.16	0	0.00	0.00
RNA metabolism protein	91	0.04	4	0.04	0.92	0	0.00	0.00
scaffold/adaptor protein	5	0.00	0	0.00	0.00	1	0.02	9.81
storage protein	1	0.00	0	0.00	0.00	0	0.00	0.00
structural protein	11	0.01	0	0.00	0.00	0	0.00	0.00
transfer/carrier protein	7	0.00	1	0.01	2.97	0	0.00	0.00
translational protein	101	0.05	16	0.16	3.30	0	0.00	0.00
transmembrane signal receptor	25	0.01	0	0.00	0.00	1	0.02	1.96
transporter	340	0.16	10	0.10	0.61	16	0.38	2.31
viral or transposable element protein	65	0.03	0	0.00	0.00	0	0.00	0.00

Table S8. Log_2 fold change and adjusted p values for RNA-seq, Ribo-seq, and TE for select genes with differential TE.

Gene name	Function	RNA-seq fold change	RNA-seq p-value	Ribo-seq fold change	Ribo-seq p-value	TE fold change	TE p-value
**HVO_RS04895**	DUF6789 family protein	1.49	5.33E-07	3.37	2.15E-16	1.87	0.001
**HVO_RS13250**	catalase/peroxidase HPI (KatG)	2.48	2.92E-27	1.49	1.34E-10	-1.01	0.01
**HVO_RS07285**	DNA starvation/stationary phase protection protein DpsA	2.79	3.95E-21	1.18	0.001	-1.63	0.007

Table S9. TPMs for all sRNAs. See xlsx file.

Table S10. REPARATION summary statistics. See xlsx file.

Table S11. Novel genes predicted by REPARATION. See xlsx file.

Table S12. List of ORFs encoded by sRNAs as predicted by ORFfinder. See xlsx file.

Table S13: Protein detection by MS. See xlsx file.

Table S14. IntaRNA output for sRNA_58. See xlsx file.

Table S15. IntaRNA output for sRNA_77. See xlsx file.

Table S16. IntaRNA output for sRNA_83. See xlsx file.

Table S17. Gene functions from NCBI for sRNA_24 targets.

Gene Name	Gene function
**HVO_RS03020**	IclR family transcriptional regulator
**HVO_RS03025**	galactonate dehydratase
**HVO_RS19875**	class II aldolase/adducin family protein
**HVO_RS03035**	anaerobic glycerol-3-phosphate dehydrogenase subunit GlpA
**HVO_RS03040**	glycerol-3-phosphate dehydrogenase subunit GlpB
**HVO_RS03045**	anaerobic glycerol-3-phosphate dehydrogenase subunit GlpC
**HVO_RS03050**	heme-binding protein
**HVO_RS03055**	trehalose utilization protein ThuA
**HVO_RS03060**	inositol monophosphatase

Table S18. Software version and commands used for data analysis. See xlsx file.

Table S19. Statistical summary for the RNA-seq data analyses. See xlsx file.

Table S20. Statistical summary for Ribo-seq data analyses. See xlsx file.

Table S21. Statistical summary for RNA-seq data analyses for sRNA knockout strains. See xlsx file.

Table S22. Statistical summary of MS data analysis.

Sample	Proteins	Peptides	PSMs	Total Spectral Count (MS/MS)	Match Rate
**Control_A**	1744	14225	25732	81064	31.74
**Control_B**	1586	12180	22673	83906	27.02
**Control_C**	1853	16100	29626	84106	35.22
**Oxi_A**	1718	14103	25345	79987	31.69
**Oxi_B**	2090	19584	40301	98215	41.03
**Oxi_C**	1985	18702	34679	90709	38.23

Table S23. qPCR Primers

Primer Name	5' to 3' sequence
**sRNA_24 F**	GAACATCCAGTTGAGGGTCCTCCC
**sRNA_24 R**	TCCGTCTCATGCCGCACAGTAG
**HVO_RS03035 F**	TCAGAGACGCCATCGACCAAG
**HVO_RS03035 R**	ATAGAGTTCGGCTCCCAGCC
**HVO_RS03040 F**	TGAAGCCGACCGCACGATACC
**HVO_RS03040 R**	TCAAGCGAGTCCGCGAGCATG
**HVO_RS03045 F**	TACGATACCGGCTCGGAACTG
**HVO_RS03045 R**	GGAAACTCCTCGTCCACCTCAG
**HVO_RS03050 F**	CTCGCCTCCGTCAACATCTC
**HVO_RS03050 R**	CAGCCCGTACAGCGAGTTC
**HVO_RS19875 F**	GAGTTCGGCCGTGAGATGCTCC
**HVO_RS19875 R**	CCTCGGATTCGATCTCGTCGTAGG
**RpoA1 F (housekeeping)**	CCTGAAGGTGACAGAGAAGAACTG
**RpoA1 R (housekeeping)**	CGTGCGCCATGATGGA

FIGURES S1 TO S9

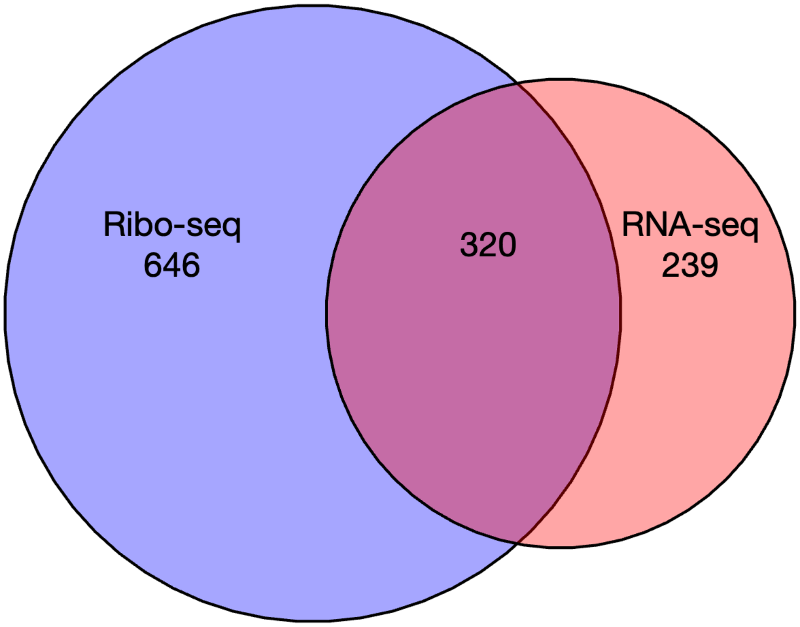

Fig. S1. Venn diagram showing overlap of genes with differential expression and differential ribosome occupancy.

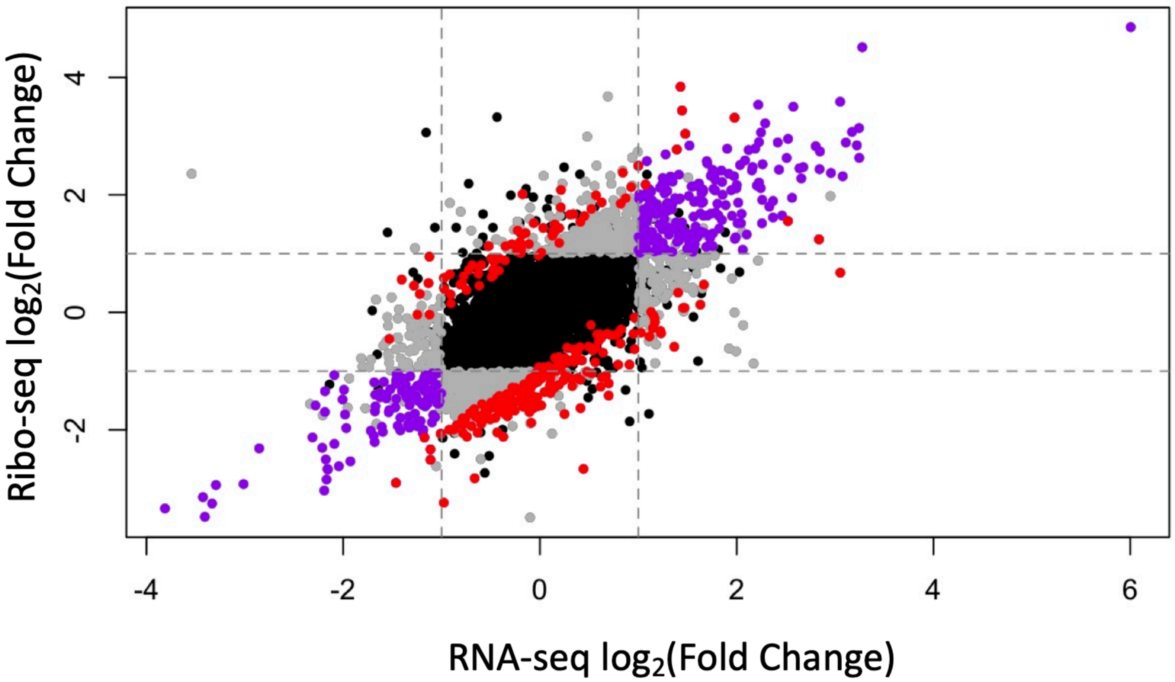

Fig S2. Scatterplot comparing $\log_2$ fold change for transcript abundances and ribosome occupancies. Genes with significant differential expression (p-value <0.05 and absolute $\log_2$ fold change >1) in RNA-seq or Ribo-seq data are shown in gray, while those with differential expression in both are shown in purple. Genes with differential TE are shown in red. All other genes (non-significant) are shown in black.

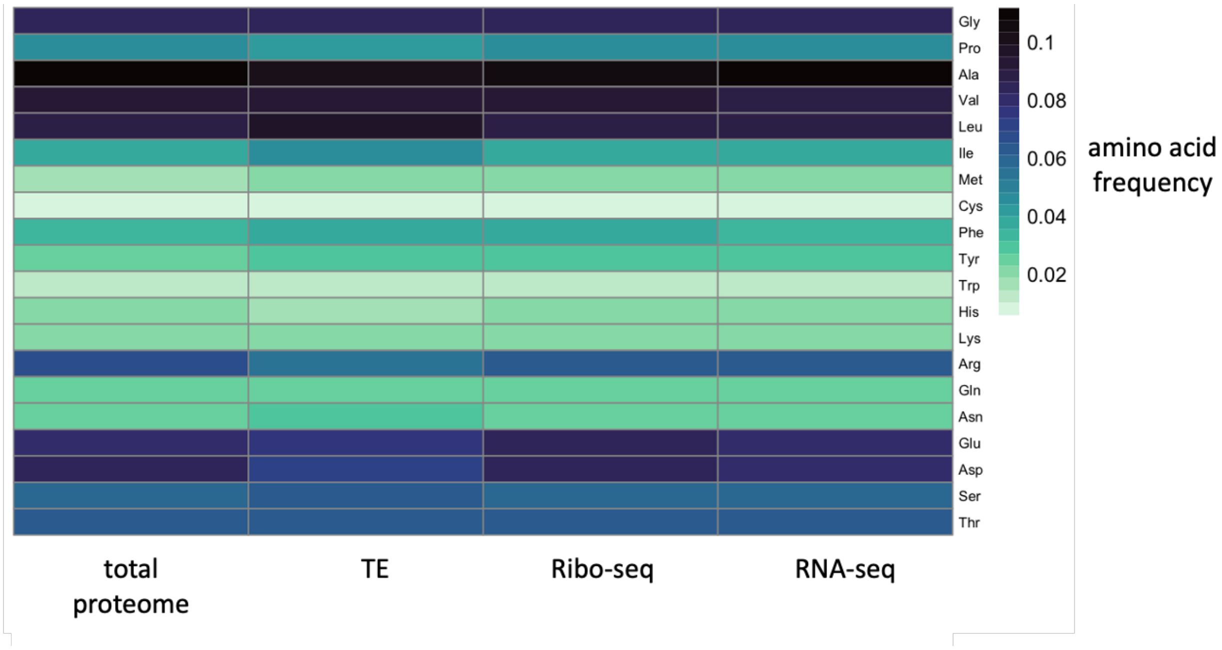

Fig. S3. Amino acid frequencies for genes with differential TE, ribosome occupancy (Ribo-seq), and expression (RNA-seq). Darker colors indicate higher frequency.

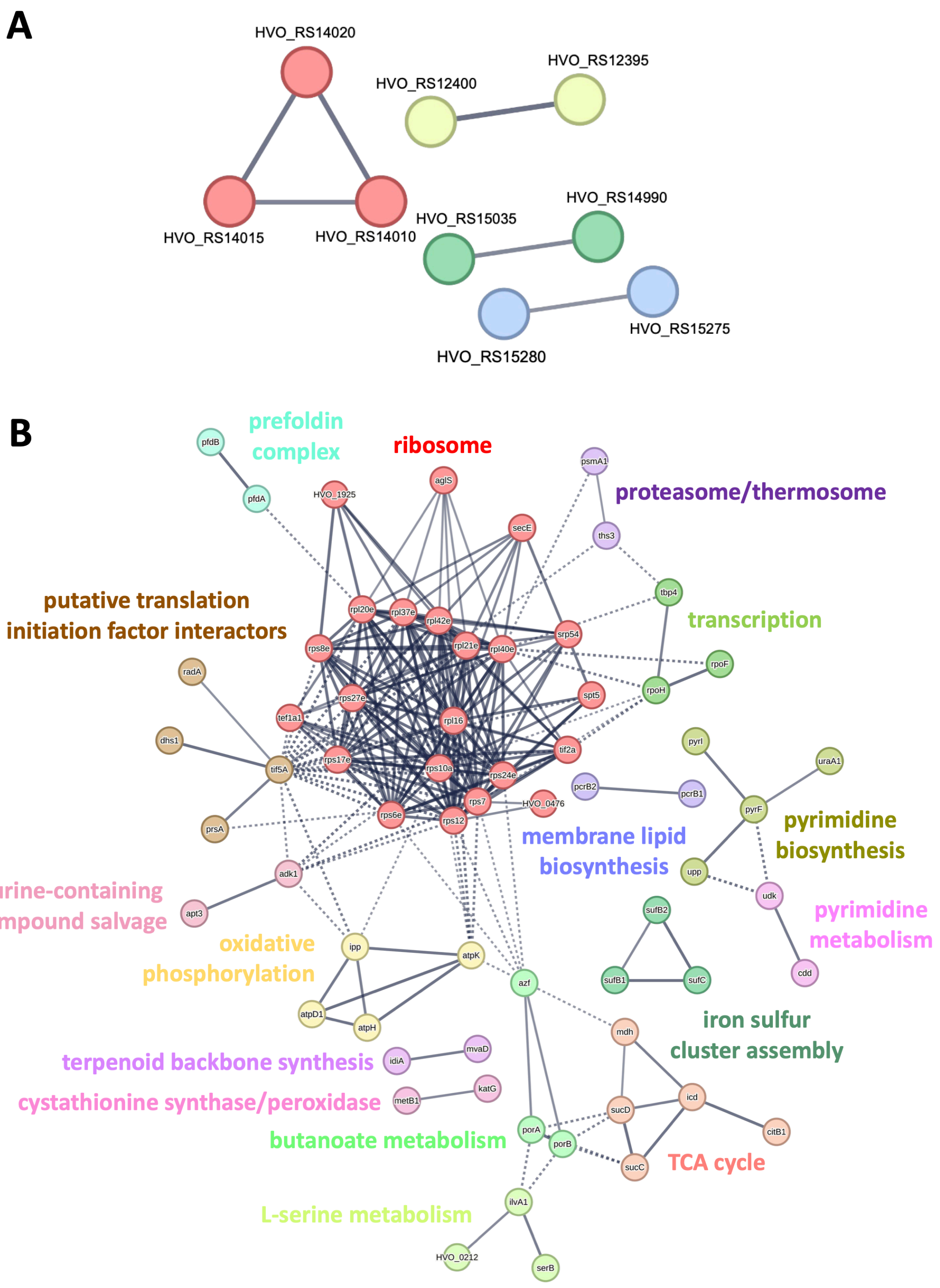

Fig. S4. STRING networks depicting predicted interactions for genes with upregulated TE (A) and downregulated TE (B) during oxidative stress, colored by cluster. Genes with no predicted interactions are not shown. Nodes depict individual genes, with predicted interactions as edges. Edge thickness indicates increasing interaction confidence, where thinner edges have a confidence score of at least 0.7 and thicker edges have a confidence score of at least 0.9. Dotted lines indicate edges between clusters. Genes with no predicted interactions are not shown. Node color indicates functional clusters.

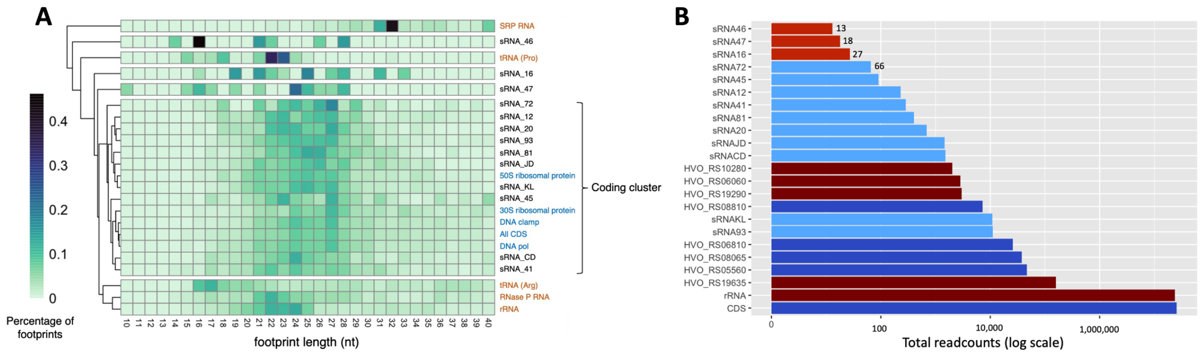

Fig. S5. Determination of clustering read count threshold. A. Clustered heatmap of ribosome footprint length distributions under control conditions for sRNAs with annotated smORFs. Three sRNAs did not cluster with coding genes B. Total read counts for sRNAs with annotated smORFs under control conditions. sRNAs with the lowest read counts did not cluster with coding genes. Blue indicates genes contained in the coding cluster, while red indicates genes not contained in the coding cluster. Darker colors indicate known coding and noncoding genes, while lighter colors show sRNAs with annotated smORFs.

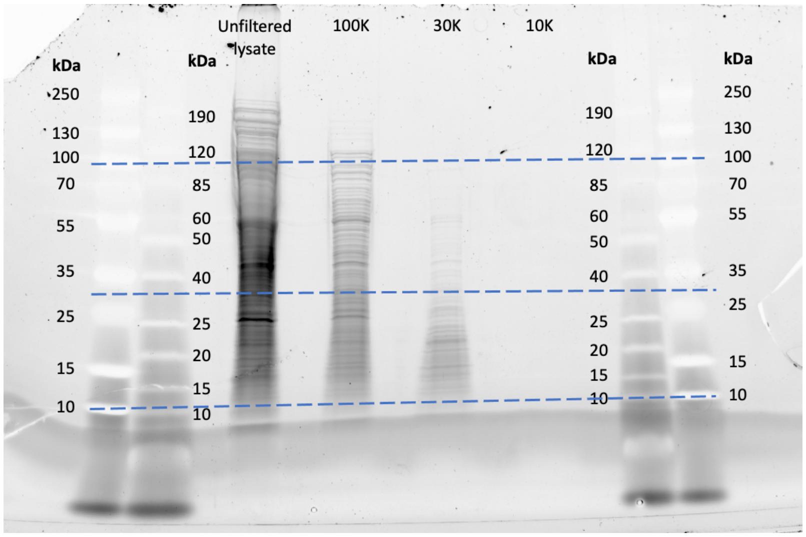

Fig. S6. SDS-PAGE of cell lysate at each filtering step prior to mass spectrometry. Gel was stained with SYPRO Ruby to visualize proteins.

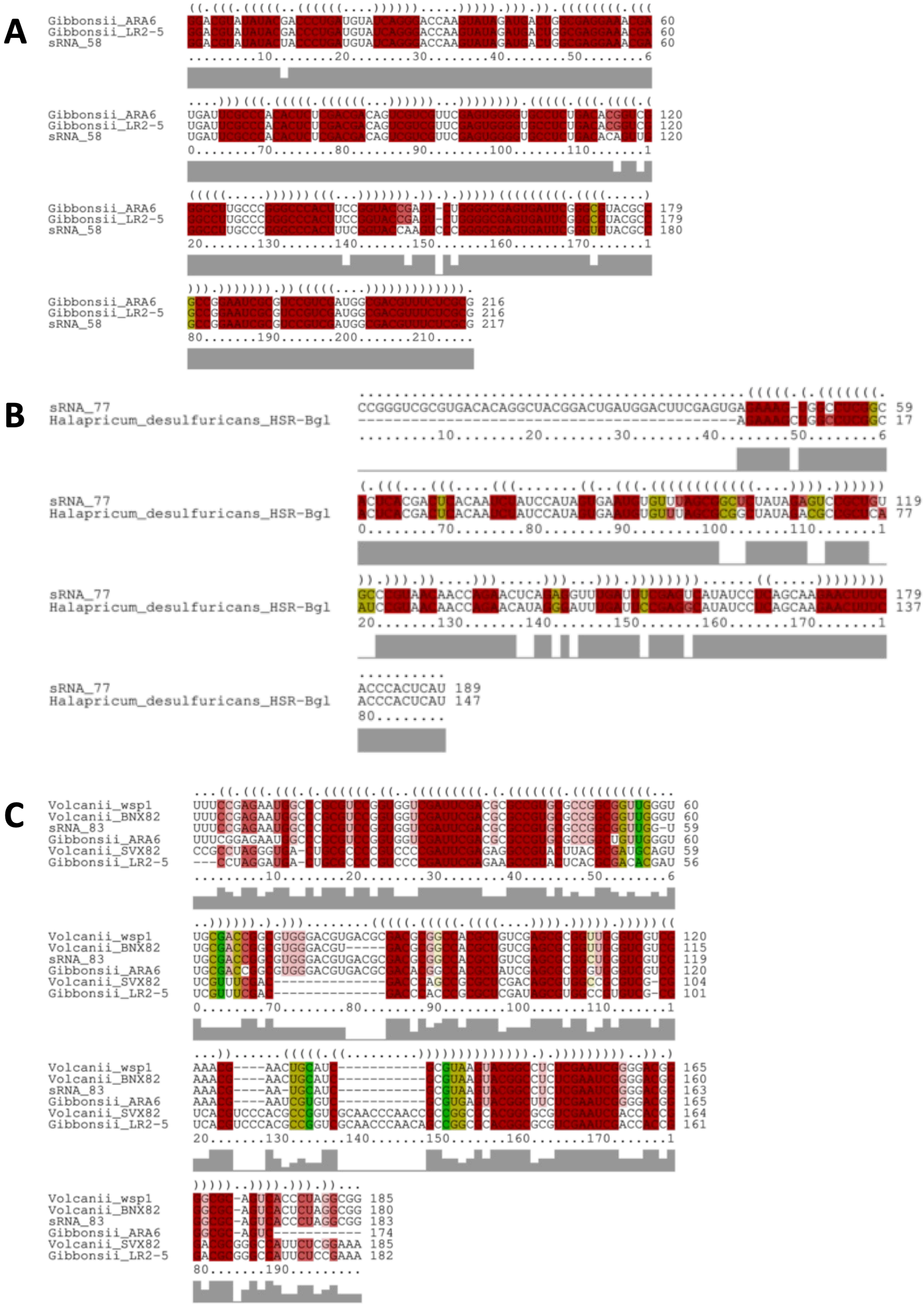

Fig. S7. Alignments for sRNA_58 (A), sRNA_77 (B), and sRNA_83 (C) were generated using LocARNA. Colored bases indicate sequence and structural conservation of compatible base pairs within the predicted structure. Color corresponds to number of base pair types (red: 1, yellow: 2, green: 3) with red being the most highly conserved and green the least conserved. Color saturation decreases with the number of incompatible base pairs.

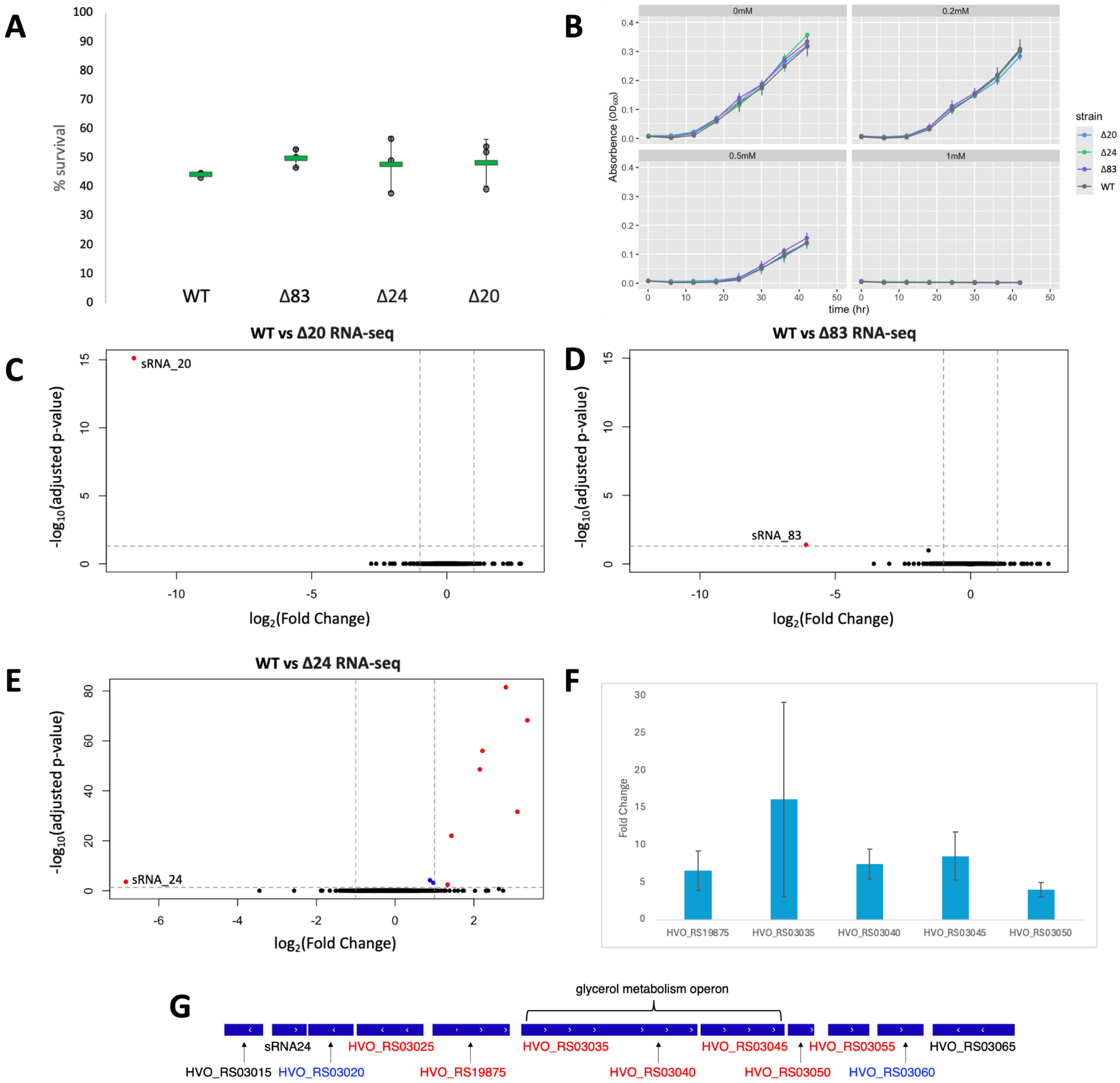

Fig. S8. Phenotyping sRNA knockout strains. A. Percent survival was determined for each sRNA knockout strain by calculating CFUs/ml before and after oxidative stress treatment. Gray points indicate values for individual replicates, with the average shown in green. Error bars indicate standard deviation. B. Growth curves over 42 hours with varying doses of hydrogen peroxide. Absorbance values are the average of 3 biological replicates. Error bars show standard deviation. C-E. Volcano plots comparing p-values and fold change for WT and sRNA knockout strains. Red points highlight transcripts with an adjusted p-value <0.05 and absolute $\log_2$ fold change >1. Blue points highlight transcripts with an adjusted p-value <0.05. Fold change and p-values were calculated using DESeq2. F. qPCR results for sRNA_24 knockout strain compared to WT. Both strains were exposed to oxidative stress. Error bars show standard error. RpoA1 was used as the housekeeping gene. G. sRNA_24 targets are the nine genes downstream of the sRNA. Genes labeled in red indicate transcripts with an adjusted p-value <0.05 and absolute $\log_2$ fold change >1, while those with gene names in blue indicate transcripts with an adjusted p-value <0.05.

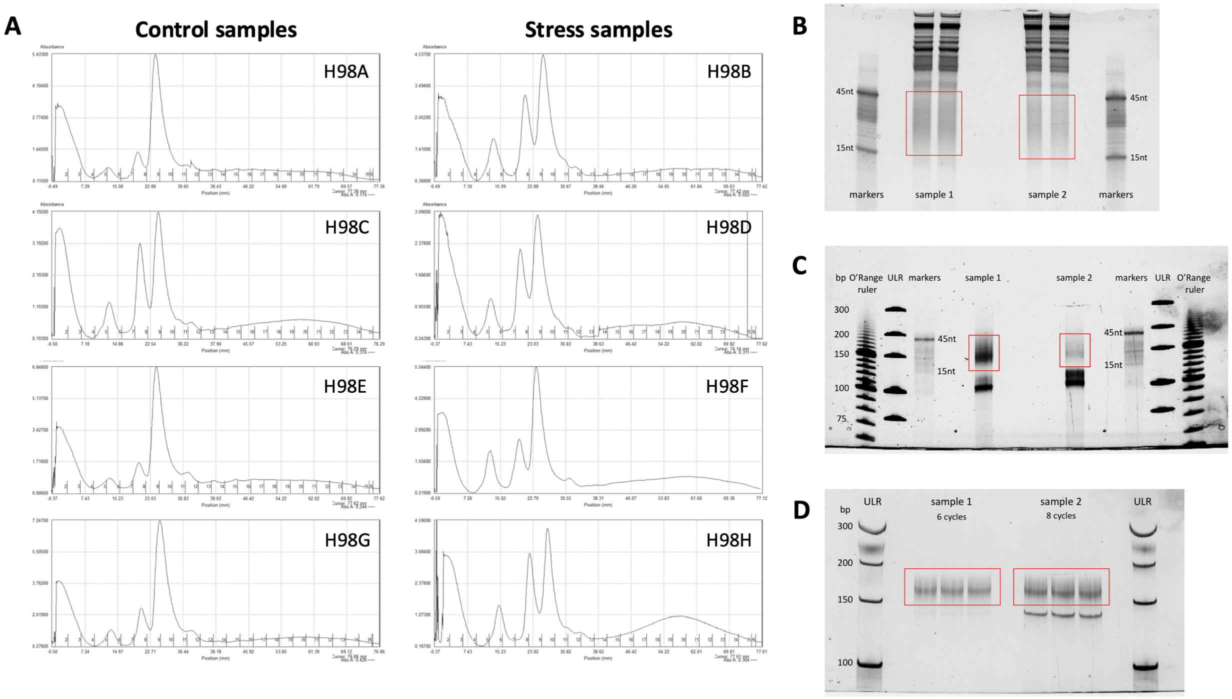

Fig. S9. Ribosome profiling library preparation. A. Traces from fractionation of monosomes for ribosome profiling libraries. B. Representative gel for size selection of ribosome library construction. C. Representative gel for reverse transcription steps of ribosome library construction. D. Representative gel for preparatory PCR step of ribosome library construction.
